# Seizing pandemic lessons: a call to reinvent public health and healthcare policy planning in post-COVID-19 Hong Kong

**DOI:** 10.3389/fpubh.2024.1378148

**Published:** 2024-03-15

**Authors:** Wengtong Chan, Chonin Cheang

**Affiliations:** Faculty of Health Sciences, University of Macau, Macau, Macao SAR, China

**Keywords:** public health, healthcare policy, post-COVID-19, Hong Kong, policy

The COVID-19 pandemic has been an unrelenting test of healthcare systems worldwide, pushing them to their limits and exposing underlying weaknesses. Among those tested, Hong Kong has had its share of trials but has also emerged with crucial insights from its fight against the virus. As we grapple with the current crisis and anticipate a post-COVID-19 future, it's crucial that these insights are utilized to not only inform but actively shape the planning and implementation of public health and healthcare policies.

A key lesson from the pandemic is the indispensable role of an effective and rapid response system. With the world caught in the throes of an unfolding health crisis, Hong Kong's proactive approach—early recognition of the virus threat and swift implementation of stringent measures, such as testing, contact tracing, quarantine, and border control—has been instrumental in managing the pandemic (1). This experience sheds light on the urgent need for a robust surveillance and response infrastructure, ready to react at a moment's notice to future health emergencies. It's a call for all stakeholders to prioritize the development of such infrastructure, enabling a faster and more efficient response to crises. In addition, we propose integrating the insights gained from Hong Kong's pandemic response within the framework of Health System Resilience (HSR) (2). This approach allows us to examine the city's response mechanisms not just as effective strategies but as components of a resilient health system capable of anticipating, responding to, and recovering from health crises.

Similarly, the pandemic has underscored the importance of a resilient healthcare system. The surge in COVID-19 cases strained Hong Kong's hospitals almost to their breaking point, revealing gaps in capacity and resources (3). The Center for Health Protection (CHP) of the Department of Health (DH) has provided showcases the significant impact of COVID-19 on Hong Kong, with a total of 1,226,467 cases detected via nucleic acid tests and 1,880,112 cases identified through Rapid Antigen Tests (RATs) up to January 29, 2023 (4). This was a wake-up call, demonstrating the urgent need for greater investment in healthcare infrastructure. To build a resilient healthcare system, we need to expand hospital bed capacity, reinforce our healthcare workforce, and ensure a steady supply of personal protective equipment. It's not enough to meet the needs of the present; we must also anticipate and prepare for future crises.

The pandemic has also driven home the importance of public health communication. The successful management of the pandemic in Hong Kong was due in part to effective risk communication, which ensured public understanding and adherence to preventive measures (5). Going forward, it is vital to enhance communication strategies, with an aim to ensure accurate, timely, and transparent information sharing. Similarly, it's essential to acknowledge critiques and opposing viewpoints to foster a balanced discourse. We have to focus on regarding privacy in digital contact tracing, the sustainability of strict border controls, and the risk of healthcare system overload, presenting a nuanced analysis of these issues.

Equally important is the recognition of the impact of social determinants on health. Disparities in COVID-19 outcomes in Hong Kong are tied to factors such as housing conditions and occupational risks (6). This highlights the need for a more comprehensive approach to health policy planning, one that considers socio-economic factors and is committed to addressing health inequalities. It's time to acknowledge that health is more than healthcare; it's about creating conditions that enable all individuals to lead healthy lives.

The pandemic has brought to the fore the importance of interdisciplinary collaboration. The fight against COVID-19 required a concerted effort from various sectors—from healthcare and public health to technology and logistics. Future policies should encourage greater interdisciplinary cooperation and harness the potential of digital health technologies for disease surveillance, contact tracing, and health service delivery (7), such as the comparison of infection rates, mortality rates, and the efficiency of contact tracing systems relative to other regions. Because the lessons learned from Hong Kong's experience are not isolated but resonate with global challenges in public health. The early experiences of Hong Kong, Singapore, and Japan serve as important case studies for other countries and regions, demonstrating the value of preparedness, rapid response, and the adaptability of public health systems in the face of emerging infectious diseases. By comparing and contrasting with other regions, we can explore the universality of these insights and their potential to inform global health policy. This comparison will highlight the adaptability and scalability of Hong Kong's strategies in different socio-economic contexts.

The COVID-19 pandemic, while devastating, has provided invaluable lessons that can transform public health and healthcare policies in post-pandemic Hong Kong. By harnessing these insights—fortifying response systems, investing in healthcare infrastructure, refining communication strategies, considering social determinants of health, and fostering interdisciplinary collaboration—Hong Kong has the opportunity to not only prepare for future health emergencies but also to build a healthier, more equitable, and resilient society.

## Author contributions

WC: Writing – original draft, Writing – review & editing. CC: Writing – original draft, Writing – review & editing.
